# Biomarker-Directed Therapy in Black and White Men With Metastatic Castration-Resistant Prostate Cancer

**DOI:** 10.1001/jamanetworkopen.2023.34208

**Published:** 2023-09-18

**Authors:** Clara Hwang, Nicholas C. Henderson, Shih-Chun Chu, Brandon Holland, Frank C. Cackowski, Amanda Pilling, Albert Jang, Shoshana Rothstein, Matthew Labriola, Joseph J. Park, Alyssa Ghose, Mehmet A. Bilen, Seema Mustafa, Deepak Kilari, Michael J. Pierro, Bicky Thapa, Abhishek Tripathi, Rohan Garje, Aditya Ravindra, Vadim S. Koshkin, Erik Hernandez, Michael T. Schweizer, Andrew J. Armstrong, Rana R. McKay, Tanya B. Dorff, Ajjai S. Alva, Pedro C. Barata

**Affiliations:** 1 Henry Ford Health, Detroit, Michigan; 2 University of Michigan, Ann Arbor, Michigan; 3 Wayne State University School of Medicine, Detroit, Michigan; 4 Karmanos Cancer Institute, Detroit, Michigan; 5 Tulane University, New Orleans, Louisiana; 6 Division of Medical Oncology, Department of Medicine, Duke Cancer Institute Center for Prostate and Urologic Cancer, Duke University, Durham, North Carolina; 7 Emory University, Atlanta, Georgia; 8 Medical College of Wisconsin, Milwaukee, Wisconsin; 9 University of Oklahoma, Oklahoma City, Oklahoma; 10 University of Iowa, Iowa City, Iowa; 11 University of California San Francisco, San Francisco, California; 12 University of Washington, Seattle, Washington; 13 University of California San Diego, La Jolla, California; 14 City of Hope, Duarte, California; 15 University Hospitals Seidman Cancer Center, Cleveland, Ohio

## Abstract

**Importance:**

Black men have higher incidence and mortality from prostate cancer. Whether precision oncology disparities affect Black men with metastatic castration-resistant prostate cancer (mCRPC) is unknown.

**Objective:**

To compare precision medicine data and outcomes between Black and White men with mCRPC.

**Design, Setting, and Participants:**

This retrospective cohort study used data collected by the Prostate Cancer Precision Medicine Multi-Institutional Collaborative Effort (PROMISE) consortium, a multi-institutional registry with linked clinicogenomic data, from April 2020 to December 2021. Participants included Black and White patients with mCRPC with molecular data. Data were analyzed from December 2021 to May 2023.

**Exposures:**

Database-reported race and ethnicity.

**Main Outcomes and Measures:**

The primary outcome was the frequency of actionable molecular data, defined as the presence of mismatch repair deficiency (MMRD) or high microsatellite instability (MSI-H), homologous recombination repair deficiency, or tumor mutational burden of 10 mutations per megabase or greater. Secondary outcomes included the frequency of other alterations, the type and timing of genomic testing performed, and use of targeted therapy. Efficacy outcomes were prostate-specific antigen response rate, site-reported radiographic response, and overall survival.

**Results:**

A total of 962 eligible patients with mCRPC were identified, including 204 Black patients (21.2%; median [IQR] age at diagnosis, 61 [55-67] years; 131 patients [64.2%] with Gleason scores 8-10; 92 patients [45.1%] with de novo metastatic disease) and 758 White patients (78.8%; median [IQR] age, 63 [57-69] years; 445 patients [58.7%] with Gleason scores 8-10; 310 patients [40.9%] with de novo metastatic disease). Median (IQR) follow-up from mCRPC was 26.6 (14.2-44.7) months. Blood-based molecular testing was more common in Black men (111 men [48.7%]) than White men (317 men [36.4%]; *P* < .001). Rates of actionable alterations were similar between groups (65 Black men [32.8%]; 215 White men [29.1%]; *P* = .35), but MMRD or MSI-H was more common in Black men (18 men [9.1]) than White men (36 men [4.9%]; *P* = .04). *PTEN* alterations were less frequent in Black men than White men (31 men [15.7%] vs 194 men [26.3%]; *P* = .003), as were *TMPRSS* alterations (14 men [7.1%] vs 155 men [21.0%]; *P* < .001). No other differences were seen in the 15 most frequently altered genes, including *TP53*, *AR*, *CDK12*, *RB1*, and *PIK3CA*. Matched targeted therapy was given less frequently in Black men than White men (22 men [33.5%] vs 115 men [53.5%]; *P* = .008). There were no differences in response to targeted therapy or survival between the two cohorts.

**Conclusions and Relevance:**

This cohort study of men with mCRPC found higher frequency of MMRD or MSI-H and lower frequency of *PTEN* and *TMPRSS* alterations in Black men compared with White men. Although Black men received targeted therapy less frequently than White men, no differences were observed in clinical outcomes.

## Key Points

**Question:**

Do disparities exist in the application of precision medicine for Black and White men with metastatic prostate cancer?

**Findings:**

In this cohort study of 962 men with metastatic castration-resistant prostate cancer, mismatch repair deficiency or microsatellite instability-high was significantly more frequent in Black men than White men. However, Black men were significantly less likely to receive molecularly matched targeted therapy than White men.

**Meaning:**

These findings suggest that although precision medicine in metastatic prostate cancer has become more common, opportunities remain to improve access to precision medicine to benefit Black men with prostate cancer.

## Teaser

This cohort study compares the use of precision medicine data and outcomes among Black and White men with metastatic castration-resistant prostate cancer.

## Introduction

Black men are disproportionately affected by prostate cancer and have a higher risk of dying from prostate cancer compared with White men in the United States.^1^ These prostate cancer disparities have been attributed in part to barriers to accessing care.^1,2,3^ Furthermore, environmental factors can interact with genetic factors to induce adverse tumor biology.^4^ Notably, outcomes of Black men within equal-access health care systems, such as in the Veterans Health Administration, show similar or higher prostate cancer–specific survival compared with White men.^5^ Similarly, the efficacy of sipuleucel-T, docetaxel, and novel hormonal therapies in Black patients with advanced prostate cancer is equivalent if not superior to that in White patients.^6,7,8,9^

Recently, molecular profiling has identified biomarkers associated with response to targeted therapies in prostate cancer. For example, homologous recombination repair defects (HRRDs) are associated with response to poly–adenosine diphosphate-ribose polymerase (PARP) inhibitors^10,11,12^ and to DNA-damaging therapies agents, such as platinum chemotherapy.^13,14,15,16^ Other examples of molecular alterations associated with response include mismatch repair deficiency (MMRD) and tumors with high microsatellite instability (MSI-H).^17,18^

Because Black men have been underrepresented in prostate cancer molecular profiling studies,^19,20^ the underlying tumor genomic landscape for Black men is not entirely known. Since molecular profiling is becoming increasingly important in metastatic prostate cancer, we aimed to investigate the proportion of Black and White men with actionable molecular data and access to targeted therapies for these 2 groups in a contemporary, clinical-genomic database.

## Methods

This cohort study was either exempt from institutional review board (IRB) review or IRB-approved at all participating centers, per individual institutional policy. The University of Michigan IRB determined that informed consent was not required because no identifiable patient information was collected. Reporting follows the Strengthening the Reporting of Observational Studies in Epidemiology (STROBE) reporting guideline.

### Study Design and Patient Population

The Prostate Cancer Precision Medicine Multi-Institutional Collaborative Effort (PROMISE) is a consortium of academic cancer centers who have established a large, diverse, inclusive, and well-annotated repository of deidentified genomic and clinical data for men with advanced prostate cancer.^21^ All patients have undergone germline or somatic molecular profiling, including but not limited to 1 or more of the following blood or tissue-based assays: tumor DNA sequencing (eg, cell-free circulating tumor DNA or tumor tissue sequencing), germline DNA sequencing, or transcript profiling studies. Comprehensive clinical data are collected from the time of initial patient diagnosis to time of last follow-up or death. Data are deidentified locally and submitted to a secure database maintained at the University of Michigan. Data collection began April 2020. Data cutoff for this analysis was December 12, 2021. For this analysis, patients were required to have a diagnosis of metastatic castration-resistant prostate cancer (mCRPC) and evaluable race and ethnicity information. Two cohorts of patients were defined based on race and ethnicity: non-Hispanic Black (hereafter, *Black*) and non-Hispanic White (hereafter, *White*).

### Data Elements and Outcome Definitions

Data elements included demographics (including race and ethnicity, as captured by the PROMISE database survey instrument) and clinical and disease characteristics. Prostate-specific antigen (PSA) status was collected from the first systemic therapy given for mCRPC. Molecular testing variables included specimen source, type and timing of molecular testing performed, and reported molecular aberrations.

The primary outcome was the proportion of Black and White men with actionable molecular data. Actionable molecular data was defined as the presence of MMRD or MSI-H, HRRD, or high tumor mutational burden (TMB-H) of 10 mutations per megabase or greater. The presence of any of the following molecular alterations was sufficient for inclusion in the MMRD or MSI-H cohort: MSI; loss of MSH2, MSH3, MSH6, MLH1, MLH3, PMS1, and PMS2 on immunohistochemistry; loss of function of *MSH2*, *MSH3*, *MSH6*, *MLH1*, *MLH3*, *PMS1*, and *PMS2* on somatic or germline sequencing; or *MLH1* promoter hypermethylation. HRRD was defined as a pathogenic alteration in any of the following genes: *BRCA1*, *BRCA2*, *ATM*, *BRIP1*, *BARD1*, *CDK12*, *CHEK1*, *CHEK2*, *FANCL*, *PALB2*, *PPP2R2A*, *RAD51B*, *RAD51C*, *RAD51D*, or *RAD54L*. For patients with more than 1 test result, the presence of an actionable alteration on any test result was used for categorization.

Secondary outcomes included the proportion of Black and White men with other molecular alterations, the type and timing of molecular testing performed, and the use of biomarker-directed therapy. Biomarker-directed therapy was defined as checkpoint inhibitor immunotherapy for MMRD or MSI-H, PARP-inhibitor or platinum-based therapy for HRRD, and checkpoint inhibitor immunotherapy for TMB-H.

Disease-related outcomes included PSA declines, site-reported best radiographic response, and overall survival (OS). PSA declines were calculated as the percentage decline from baseline to nadir PSA, and PSA response (yes vs no) was categorized as at least 50% PSA decline. Response rates were calculated as the percentage of patients with either complete or partial radiographic response (site-reported) for each category of therapy. Survival was calculated from the time of site-reported mCRPC until death or censored at date of last contact. OS probabilities for Black and White cohorts were estimated with the Kaplan-Meier method.

Patient characteristics were summarized using the median and IQR for noncategorical variables, and categorical variables were described with the proportions in each category. Differences between the Black and White cohorts for categorical or binary variables were evaluated using χ^2^ tests, and 95% CIs for proportions were constructed using the Wilson method. Overall survival probabilities for the Black and White cohorts were estimated using the Kaplan-Meier method, and differences in OS across groups were evaluated using the log-rank test. Statistical significance was defined as 2-sided *P* < .05. Analyses were conducted using R statistical software version 4.1.2 (R Project for Statistical Computing). Data were analyzed from December 2021 to May 2023.

## Results

### Baseline Characteristics

Of 1619 individuals in the overall database, a total of 962 patients with mCRPC met inclusion criteria (eFigure 1 in the Supplement), including 204 Black patients (21.2%; median [IQR] age at diagnosis, 61 [55-67] years; 131 patients [64.2%] with Gleason scores 8-10; 92 patients [45.1%] with de novo metastatic disease) and 758 White patients (78.8%; median [IQR] age, 63 [57-69] years; 445 patients [58.7%] with Gleason scores 8-10; 310 patients [40.9%] with de novo metastatic disease). Baseline characteristics are summarized in Table 1. Fewer Black men received prior definitive therapy (prostatectomy: 60 Black men [29.4%]; 308 White men [40.6%]; *P* = .004; radiation: 83 Black men [40.7%]; 356 White men [47.0%]; *P* = .08). Median (IQR) PSA at the time of mCRPC was 19.8 (5.85-78.0) ng/mL for Black men and 10.2 (3.0-32.2) ng/mL for White men (to convert to micrograms per liter, multiply by 1). The top 5 institutions contributing to the Black cohort were Henry Ford, Emory, Karmanos, Duke, and Tulane (148 patients [50% of the total Black cohort]).

**Table 1.** Baseline Patient and Disease Characteristics of the Study Cohort

Characteristic	Participants, No. (%) (N = 962)	
	Black (n = 204)	White (n = 758)
Age at diagnosis, median (range) [IQR], y	61 (33-91) [55-67]	63 (38-93) [57-69]
Marital status		
Single	43 (21.1)	59 (7.8)
Married	119 (58.3)	577 (76.1)
Separated or divorced	22(10.8)	44 (5.8)
Widowed	5 (2.5)	31 (4.1)
Unknown	15 (7.4)	47 (6.2)
Smoking status		
Never	84 (41.2)	405 (53.4)
Yes, current	31 (15.2)	46 (6.1)
Yes, former	87 (42.6)	288 (38.0)
Smokeless tobacco	0 (0.0)	8 (1.1)
Unknown	2 (1.0)	11 (1.5)
Treatment site^a^		
University of Michigan	14 (6.9)	120 (15.8)
Emory	41 (20.1)	41 (5.4)
UCSD	2 (1.0)	46 (6.1)
University of Washington	5 (2.5)	75 (9.9)
Duke	22 (10.8)	97 (12.8)
MCW	14 (6.9)	72 (9.5)
City of Hope	7 (3.4)	66 (8.7)
UCSF	2 (1.0)	45 (5.9)
University of Oklahoma	11 (5.4)	56 (7.4)
Karmanos	23 (11.3)	14 (1.8)
Henry Ford	46 (22.5)	33 (4.4)
Tulane	16 (7.8)	50 (6.6)
University of Iowa	1 (0.5)	39 (5.1)
Nevada CCC	0	2 (0.3)
Family history of cancer		
Yes	128 (62.7)	506 (66.8)
No	71 (34.8)	226 (29.8)
Unknown	5 (2.5)	26 (3.4)
Histology		
Adenocarcinoma	182 (89.2)	672(88.7)
NEPC or SC	2 (1.0)	5 (0.7)
Other/mixed	2 (1.0)	3 (0.4)
Unknown	20 (9.8)	80 (10.6)
Gleason sum		
<6	1 (0.5)	1 (0.1)
6	6 (2.9)	30 (4.0)
7	31 (15.2)	171 (22.6)
8-10	131 (64.2)	445 (58.7)
Unknown	35 (17.2)	111 (14.6)
Prior definitive treatment		
Radical prostatectomy		
Yes	60 (29.4)	308 (40.6)
No	141 (69.1)	440 (58.0)
Unknown	3 (1.5)	10 (1.3)
Radiation therapy		
Yes	83 (40.7)	356 (47.0)
No	120 (58.8)	384 (50.7)
Unknown	1 (0.5)	18 (2.4)
Prior systemic therapies		
Adjuvant or neoadjuvant	61 (29.9)	53 (33.3)
nmCSPC	14 (6.9)	54 (7.1)
nmCRPC	7 (3.4)	20 (2.6)
mCSPC	72 (35.3)	241 (31.8)
Not reported	50 (24.5)	190 (25.1)
De novo metastatic disease		
Yes	92 (45.1)	310 (40.9)
No	107 (52.5)	430 (56.7)
Unknown	5 (2.5)	18 (2.4)
Initial PSA at stage IV at time of mCRPC, median (range) [IQR], ng/mL	19.8 (0-5000) [5.85-78.0]	10.2 (0-2672.8) [3.0-32.2]
Location of metastases at time of diagnosis		
Lymph node	69 (33.8)	227 (29.9)
Bone	74 (36.3)	244 (32.2)
Visceral	17 (8.3)	46 (6.1)
Brain or LM (CNS)	1 (0.5)	1 (0.1)
Location of metastases at time of mCRPC		
Lymph node	111 (54.4)	350 (46.2)
Bone	146 (71.6)	574 (75.7)
Visceral	44 (21.6)	146 (19.3)
Brain or LM (CNS)	2 (1.0)	5 (0.7)

Abbreviations: CCC, Comprehensive Cancer Center; CNS, central nervous system; LM, leptomeningeal; mCSPC, metastatic castration-sensitive prostate cancer; mCRPC, metastatic castration-resistant prostate cancer; MCW, Medical College of Wisconsin; NEPC, neuroendocrine prostate cancer; nmCSPC, nonmetastatic castration-sensitive prostate cancer; nmCRPC, nonmetastatic castration-resistant prostate cancer; PSA, prostate-specific antigen; SC, small cell; UCSD, University of California, San Diego; UCSF, University of California, San Francisco.

SI conversion factor: To convert PSA to micrograms per liter, multiply by 1

^a^ Proportions of patients identified as Black in our total cohort of Black patients: University of Michigan, 10.4%; Emory, 50%; UCSD, 4.2%; University of Washington, 6.3%; Duke, 18.5%; MCW, 16.3%; City of Hope, 9.5%; UCSF, 4.3%; University of Oklahoma, 16.4%; Karmanos, 62.2%; Henry Ford, 58.2%; Tulane, 24.2%; University of Iowa, 2.5%; Nevada CCC, 0%.

### Molecular Testing Characteristics

We compared molecular testing practices in the Black and White cohorts (Table 2). Most patients had molecular testing performed only once (157 Black patients [82.2%]; 562 White patients [76.7%]; *P* = .12). Patterns of serial testing are presented in eFigure 2 in Supplement 1. Somatic molecular testing was preferred in both cohorts (171 Black patients [89.5%]; 613 White patients [83.6%]; *P* = .07); germline testing was performed in 20 Black patients (10.5%) and 120 White patients (16.3%). Use of blood-based molecular testing (as opposed to tissue testing) was more common in Black men (111 patients [48.7%]) than White men (317 patients [36.4%]; *P* < .001). Molecular testing was discussed at a molecular tumor board in 18 Black patients (9.4%) and 87 White patients (11.9%) (*P* = .28). Testing was typically ordered in the mCRPC setting. The median (IQR) time from diagnosis to first molecular result was 56.3 (24.9-116.6) months for Black patients compared with 58.7 (22.3-106.8) months for White patients (*P* = .45). Other than the difference in liquid vs tissue testing, we did not find any significant differences in testing practices between the Black and White cohorts.

**Table 2.** Molecular Testing Characteristics Reported in Prostate Cancer Precision Medicine Multi-Institutional Collaborative Effort Database in Black vs White Men With mCRPC

Characteristic	Participants, No. (%)		*P* value
	Black (n = 191)	White (n = 733)	
Genomic tests performed, No.			
1	157 (82.2)	562 (76.7)	.12^a^
2	22 (11.5)	117 (16.0)	
>2	12 (6.3)	54 (7.4)	
Type of testing performed			
Somatic only	171 (89.5)	613 (83.6)	.07
Germline only	0	9 (1.2)	
Somatic and germline	20 (10.5)	111 (15.1)	
Somatic tests			
Tissue	117 (51.3)	555 (63.6)	<.001
Liquid	111 (48.7)	317 (36.4)	
Report discussed at MTB			
Yes	18 (9.4)	87 (11.9)	.28
No	96 (50.3)	323 (44.1)	
Unknown	77 (40.3)	323 (44.1)	
Setting of first test result			
CRPC	158 (84.0)	617 (86.5)	.45
CSPC	30 (16.0)	96 (13.5)	
Metastatic	181 (96.3)	685 (95.9)	.99
Nonmetastatic	7 (3.7)	29 (4.1)	

Abbreviations: CRPC, castration-resistant prostate cancer; mCRPC, metastatic castration-resistant prostate cancer; MTB, molecular tumor board; CSPC, castration-sensitive prostate cancer.

^a^ Calculated as 1 vs more than 1.

### Genomic Characterization of Tumors and Actionable Alterations

The primary objective of our study was to estimate the proportion of Black and White men with mCRPC who were reported to have actionable alterations based on MMR or MSI status, HRRD, or TMB-H. Overall, 65 Black men (32.8%) had actionable molecular data, compared with 215 White men (29.1%) (*P* = .35) (Table 3). MMRD or MSI-H was more frequent in Black men (18 patients [9.1%]) than White men (36 patients [4.9%]; *P* = .04). There were no statistically significant differences in the other categories of actionable molecular data. HRRD was identified in 46 Black men (23.2%) and 179 White men (24.2%) (*P* = .85). *BRCA2* alterations were noted in 18 Black men and 86 White men (11.6%) (*P* = .38). TMB was not universally assessed, with 77 evaluable patients (38.9%) in the Black cohort and 272 evaluable patients (36.8%) in the White cohort. Overall, 8 Black patients (10.4%) and 21 White patients (7.7%) had TMB-H.

**Table 3.** Actionable Alterations in Black vs White Men With mCRPC

Alteration	Participants, No. (%)		*P* value
	Black (n = 198)	White (n = 739)	
Actionable alteration	65 (32.8)	215 (29.1)	.35
MMRD or MSI-H	18 (9.1)	36 (4.9)	.04
HRRD	46 (23.2)	179 (24.2)	.85
TMB			
Evaluable	77 (10.4)	272 (7.7)	NA
With high TMB	8 (4)	21 (2.8)	.53

Abbreviations: HRRD, homologous recombination repair deficiency; mCRPC, metastatic castration-resistant prostate cancer; MMRD, mismatch repair deficiency; MSI-H, microsatellite instability high; NA, not applicable; TMB, tumor mutational burden.

A secondary objective was to describe the frequencies of other genomic alterations in Black and White men (eTable 1 in Supplement 1). *PTEN* was less frequently altered in Black men (31 patients [15.7%]) than White men (194 patients [26.3%]; *P* = .003). *TMPRSS* alterations were also less common in Black men (14 patients [7.1%]) than White men (155 patients [21.0%]; *P* < .001). *CCND1* alterations were more frequent in Black men (11 patients [5.6%]) than White men (17 patients [2.3%]; *P* = .03). No other significant differences were seen in the 15 most frequently altered genes, including *TP53*, *AR*, *CDK12*, *RB1*, and *PIK3CA*. Tumor suppressor coalterations indicative of aggressive variant prostate cancer (≥2 mutations in *PTEN*, *TP53*, or *RB1*)^22^ were found in 26 Black patients (13.1%) and 133 White patients (18.0%) (*P* = .13). *AR-V7* was tested infrequently, with only 9 evaluable patients in the Black cohort (4.5%) and 27 evaluable patients in the White cohort (3.7%). Of evaluable patients, *AR-V7* alterations were detected in 2 Black patients (22.2%) and 12 White patients (44.4%). An OncoPrint comparison of the Black and White cohorts is presented in eFigure 3 in Supplement 1.

### Use of Biomarker-Directed Therapies

Certain molecular alterations qualify patients for biomarker-directed therapies. We evaluated the use of biomarker-directed therapy in the MMRD or MSI-H, HRRD, and TMB-H cohorts (Table 4). For MMRD or MSI-H, 3 of 18 Black patients (16.7%), compared with 17 of 36 White patients (47.2%), were treated with pembrolizumab (*P* = .06). In the HRRD cohort, 14 of 46 eligible Black patients (30.4%) and 69 of 179 of eligible White patients (38.5%) were prescribed a PARP inhibitor (*P* = .40). Platinum chemotherapy was given in 7 of 46 eligible Black patients (15.2%) and 51 of 179 eligible White patients (28.5%) (*P* = .10). For patients with TMB-H, immunotherapy was given to 3 of 8 eligible Black patients (37.5%) and 12 of 21 eligible White patients (57.1%) (*P* = .60). Overall, biomarker-directed therapy was given to 22 of 65 eligible Black patients (33.5%) and 115 of 215 eligible White patients (53.5%) (*P* = .008).

**Table 4.** BDT in Black and White Cohorts

Therapy	Participants, No. (%)		*P* value
	Black	White	
Pembrolizumab for MMRD or MSI-H^a^	3 (16.7)	17 (47.2)	
HRRD^b^			
PARP inhibitor			.40
Any	14 (30.4)	69 (38.5)	
Olaparib	12 (26.1)	59 (33.0)	
Rucaparib	2 (4.3)	6 (3.4)	
Talazoparib	1 (2.2)	3 (1.7)	
Niraparib	0	1 (0.6)	
Platinum chemotherapy	7 (15.2)	51 (28.5)	.10
TMB high^c^			
Any immune checkpoint inhibitor	3 (37.5)	12 (57.1)	.60
Pembrolizumab	2 (25.0)	11 (52.4)	
Nivolumab	1 (12.5)	0	
Atezolizumab	0	1 (4.8)	
MMRD or MSI-H, HRRD, or TMB high^d^			
Any BDT	22 (33.5)	115 (53.5)	.008
BDT in clinical trial	2 (3.1)	14 (6.5)	.07

Abbreviations: BDT, biomarker-directed therapy; HRRD, homologous recombination repair deficiency; MMRD, mismatch repair deficiency; MSI-H, microsatellite instability high; PARP, poly–adenosine diphosphate ribose polymerase; TMB, tumor mutational burden.

^a^ Among 18 Black patients and 36 White patients.

^b^ Among 46 Black patients and 179 White patients.

^c^ Among 8 Black patients and 21 White patients.

^d^ Among 65 Black patients and 215 White patients.

When reviewing treatment patterns in the MMRD or MSI-H, HRRD, and TMB-H cohorts, we discovered the use of biomarker-directed therapy that was not approved by the US Food and Drug Administration. Therefore, we compared the use of clinical trials in the Black and White cohorts in an exploratory analysis. We found that 14 of 215 White patients (6.5%) and 2 of 65 Black patients (3.1%) received biomarker-directed therapy in a clinical trial.

### Disease-Related Outcomes

There were no differences seen in response to biomarker directed therapy (eTable 2 in Supplement 1). PSA response rates to immunotherapy were 100% (95% CI, 31.0%-100%) in the Black cohort and 66.7% (95% CI, 41.2%-85.6%) in the White cohort (*P* = .62). Radiographic response rates to immunotherapy were 50% (95% CI, 15.0%-85.0%) in the Black cohort and 61.5% (95% CI, 32.3%-84.9%) in the White cohort (*P* > .99). For PARP inhibitor therapy, the PSA response rate was 30.8% (95% CI, 10.4%-61.1%) in the Black cohort and 41.9% (95% CI, 29.7%-55.1%) in the White cohort (*P* = .66), and the radiographic response rate was 22.2% (95% CI, 3.9%-59.8%) in the Black cohort and 14.8% (95% CI, 7.4%-26.7%) in the White cohort (*P* = .93). Finally, for platinum-based chemotherapy, the PSA response rate in the Black cohort was 66.7% (95% CI, 24.1%-94.0%) and 42.0% (95% CI, 28.5%-56.7%) in the White cohort (*P* = .48). The radiographic response rate for platinum therapy was 25.0% (95% CI, 1.3%-78.1%) in the Black cohort and 31.6% (95% CI, 18.0%-48.8%) in the White cohort (*P* > .99).

Similarly, no difference was seen in survival between the Black and White cohorts. Median OS from development of mCRPC was 41.5 (95% CI, 34.7-51.3) months for Black men and 44.7 (95% CI, 41.1-51.5) months for White men (*P* = .14). No difference was seen when comparing survival outcomes for patients who received biomarker-directed therapy with those who did not (Figure). The median OS from mCRPC for White men was 44.9 (95% CI, 37.9-61.9) months with BDT and 39.8 (95% CI, 28.5 to not reported [NR]) months without BDT. The median OS from mCRPC for Black men was 46.2 (95% CI, 37.0-NR) months with BDT and 50.2 (95% CI, 24.6-NR) months without BDT.

**Figure.**
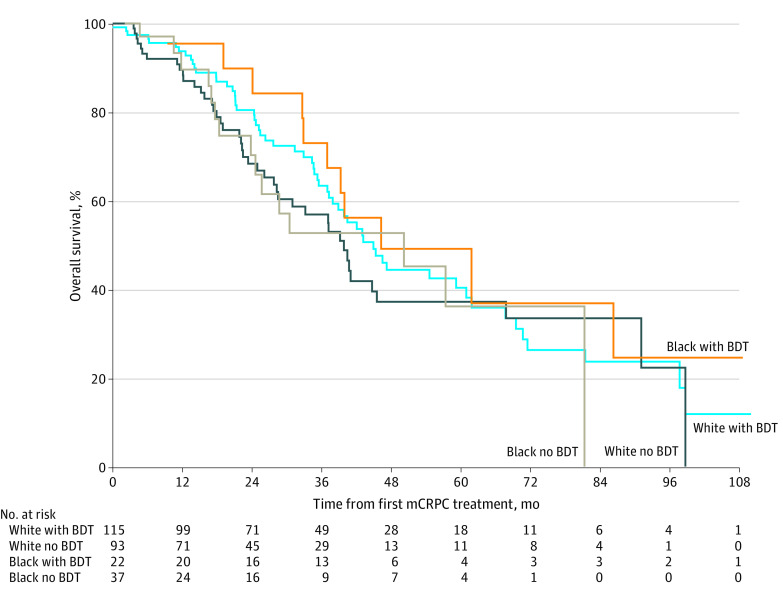
Kaplan-Meier Estimate of Overall Survival for Black and White Cohorts With and Without Biomarker-Directed Therapy (BDT) mCRPC indicates metastatic castration-resistant prostate cancer.

## Discussion

In this cohort study, we present one of the largest studies examining differences between Black and White men in a contemporary mCRPC molecular profiling cohort to our knowledge. We found no differences between the Black and White cohorts for our primary outcome, the proportion of patients with actionable molecular data; however, MMRD or MSI-H was more common in Black men. Despite this, receipt of immunotherapy in these Black men was approximately 30% lower than for White men, suggesting a barrier by race in the receipt of matched molecular immunotherapy in the US.

We identified differences in rates of MMRD or MSI-H and *PTEN* alterations between the Black and White cohorts. MMRD or MSI-H was more common in Black men, while *PTEN* alterations and *TMPRSS2* translocations were less frequent in Black men. Other studies have reported a lower frequency of *PTEN* loss and *TMPRSS2-ERG* translocations in genetic analyses of primary prostate cancers from Black men,^23,24,25^ strengthening the reliability of these findings. *PTEN* alterations have an associated poorer prognosis,^26^ but because *PTEN* loss is more frequent in White men, this difference does not explain observed disparities in prostate cancer outcomes affecting Black men.^27,28,29^

Our finding that MMRD or MSI-H was more frequent in Black men than White men is concordant with a prior report of higher rates of MMRD tumors in men of African ancestry.^30^ MMRD was associated with adverse clinical and pathologic features but unexpectedly associated with good response to hormonal therapy.^31^ MMRD or MSI-H is conventionally associated with benefit from immunotherapy,^17^ presumably related to genomic instability and the occurrence of immunogenic tumor neoantigens. Genomic instability can arise from defects in other DNA repair pathways, such as HRR. One such gene, *CDK12*, has been associated with response to immunotherapy in prostate cancer.^31,32^ Prostate cancers in Black men and men of African ancestry have been reported to have higher rates of DNA repair deficiency^33,34^ and increased TMB^35^ compared with men of European ancestry. While our study did not replicate all these findings, our data contribute to a growing body of literature pointing to greater genomic instability and possibly better outcomes for immunotherapy for Black men with prostate cancer. Increased genomic instability may be a contributing factor to the observation that tumors in Black men are associated with heightened immune activation and increased cytokine signaling.^36^ An upregulated inflammatory response and tumoral immunogenicity could also explain the greater observed survival benefit of sipuleucel-T therapy in Black men compared with White men.^6^ These data, along with the potential for durable responses, emphasize the need to screen for actionable molecular alterations that are associated with benefit from immunotherapy, especially in Black men.

Molecular testing and treatment patterns were similar between Black and White cohorts with 2 notable exceptions. Tissue-based molecular testing was less common for Black patients, and Black men were less likely to receive biomarker-directed therapy than White men. Differences in receipt of targeted therapy were not explained by a difference in clinical trial participation or discussion at molecular tumor boards. Lower use of immunotherapy and platinum chemotherapy contributed to the overall lower utilization of biomarker-directed therapy in the Black cohort. Clinical benefit from standard therapies likely influences the decision to use targeted therapy and seems justified when considering survival was the same between Black and White cohorts, with and without the use of biomarker-directed therapy. In our cohort, clinical markers of aggressive disease (eg, higher grade, presence of de novo and visceral metastases) were seen more commonly in Black men than White men. Despite this, there were no differences in OS between the Black and White cohorts. This finding is concordant with several reports of equal or better outcomes for Black patients when treated with docetaxel,^7^ sipuleucel-T,^37^ radium-223,^38^ and abiraterone,^8^ which are standard therapeutic options for men with mCRPC.

The finding that Black men were more likely to receive blood-based vs tissue-based genomic testing may be explained by the lower rates of prostatectomy in Black men^29^ leading to greater challenges with tissue-based biomarker testing. When prostatectomy tissue is not available, blood-based testing may be preferred as more convenient, less invasive, and less expensive than tissue-based testing.^39^

Multiple variables,^40,41,42,43^ including disparities in socioeconomic factors and health care access, negatively impact the care of Black men with prostate cancer. The finding that Black men were less likely to receive targeted therapies, even when undergoing molecular testing, raises the question of whether these factors played a role in this observed disparity. Resource availability at different treatment sites (proposed to be a legacy of structural racism) may influence practice patterns. Potential unconscious bias among health care practitioners is another factor that may affect the delivery of biomarker-directed therapy. Patient behavior and decision-making influence treatment delivery. Mistrust has been reported to influence cancer treatment decisions, including patients with prostate cancer undergoing genomic testing.^44^ Patient financial barriers and comorbidities may also contribute to observed treatment differences. Each of these potential variables is worthy of future study. Understanding the reasons behind observed differences in practice patterns is critical to implementing effective interventions to reduce cancer disparities. For example, community outreach or engaging trusted messengers may be used to address medical mistrust, while expanding insurance coverage, financial navigation, and assistance programs may be used to address resource disparities.

### Limitations

This study has some limitations. We did not address actionable molecular data outside of MMRD or MSI-H, TMB-H, HRRD in this study, nor did we analyze how practice patterns changed over time. The rarity of *AR-V7* testing limited our ability to compare frequencies or understand how this biomarker is used in practice. Other variables that influence whether patients receive targeted therapies include comorbidities, performance status, and socioeconomic factors, which are not captured in our database. Lack of information on insurance coverage and income constrained analysis of disparities related to social determinants of health. The PROMISE database uses database-reported race and ethnicity, which should be distinguished from genetic ancestry. Additionally, there is a potential for selection bias related to molecular testing completion as a requirement for inclusion in the PROMISE precision medicine database.

## Conclusions

In this cohort study of Black and White men with mCRPC, despite similar overall rates of actionable alterations, we found that Black men were more likely to have MMRD or MSI-H status as well as having less frequent *PTEN* alterations. We also found that Black men were less likely to receive biomarker-directed therapy. These findings underscore the need for further study for how the environment, social determinants of health, health care infrastructure, practitioner biases, and patient behavior interact to produce the cancer disparities and increased prostate cancer mortality that affect Black men.

## Notes

Supplement 1. **eFigure 1.** Participant Recruitment Flowchart **eFigure 2.** Serial NGS Testing in Black and White Cohorts **eTable 1.** Frequency of Gene Alterations in Black and White Men With mCRPC **eFigure 3.** OncoPrint of Black and White Cohorts **eTable 2.** Response Rates to Biomarker Directed Therapy

Supplement 2.
Data Sharing Statement
