# Supplementary material for: Biomarker-Directed Therapy in Black and White Men With Metastatic Castration-Resistant Prostate Cancer
Source: JAMA Netw Open. 2023 Sep 18;6(9):e2334208. doi: 10.1001/jamanetworkopen.2023.34208 (PMC10507489; doi:10.1001/jamanetworkopen.2023.34208)
Supplement: Supplement 1. — eFigure 1. Participant Recruitment Flowchart eFigure 2. Serial NGS Testing in Black and White Cohorts eTable 1. Frequency of Gene Alterations in Black and White Men With mCRPC eFigure 3. OncoPrint of Black and White Cohorts eTable 2. Response Rates to Biomarker Directed Therapy [file jamanetwopen-e2334208-s001.pdf]

Supplemental Online Content

Hwang C, Henderson NC, Chu S, et al. Biomarker-directed therapy in Black and White men with metastatic castration-resistant prostate cancer. *JAMA Netw Open*. 2023;6(9):e2334208. doi:10.1001/jamanetworkopen.2023.34208

eFigure 1. Participant Recruitment Flowchart
eFigure 2. Serial NGS Testing in Black and White Cohorts
eTable 1. Frequency of Gene Alterations in Black and White Men With mCRPC
eFigure 3. Oncoprint of Black and White Cohorts
eTable 2. Response Rates to Biomarker Directed Therapy

This supplemental material has been provided by the authors to give readers additional information about their work.

© 2023 Hwang C et al. *JAMA Network Open*

eFigure 1. Participant Recruitment Flowchart

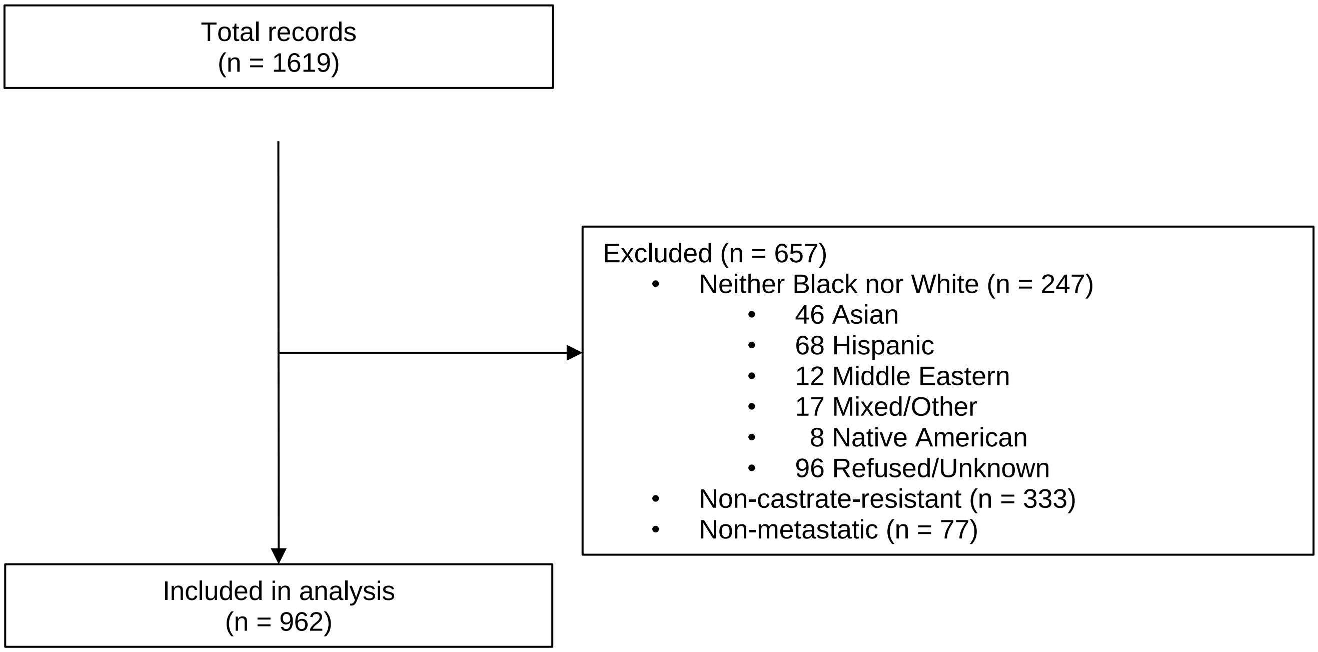

A total of 962 mCRPC patients (21.2% Black; 78.8% White) met inclusion criteria out of 1619 in the overall database. Abbreviations: mCRPC, metastatic castration-resistant prostate cancer.

© 2023 Hwang C et al. *JAMA Network Open*

eFigure 2. Serial NGS Testing in Black and White Cohorts

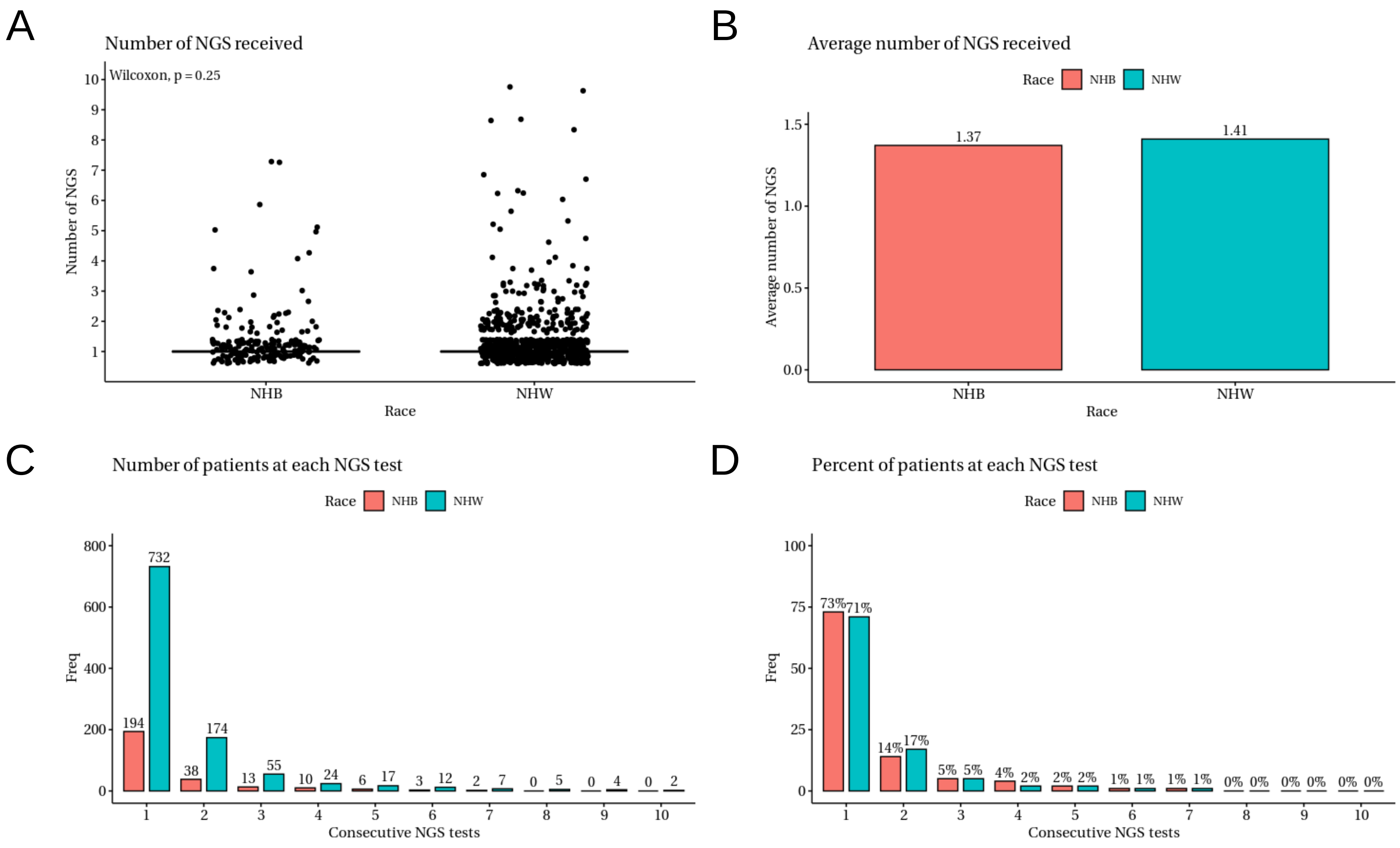

(A) Number of NGS tests performed per patient. (B) Average number of NGS tests performed in Black and White cohorts. (C) Number of patients with multiple NGS tests; X-axis represents number of NGS tests performed. (D) % of cohort with multiple NGS tests; X-axis represents number of NGS tests performed. Abbreviations: NGS, next-generation sequencing; Freq, frequency.

© 2023 Hwang C et al. *JAMA Network Open*

eTable 1. Frequency of Gene Alterations in Black and White Men With mCRPC

Gene	Black (N=198)	White (N=739)	p-value
TP53	77 (38.9%)	314 (42.5%)	0.41
AR	64 (32.3%)	280 (37.9%)	0.17
PTEN	31(15.7%)	194 (26.3%)	0.003
MYC	25 (12.6%)	66 (8.9%)	0.15
BRCA2	18 (9.1%)	86 (11.6%)	0.38
PIK3CA	18 (9.1%)	43 (5.8%)	0.13
TMPRSS2	14 (7.1%)	155 (21.0%)	< 0.001
RB1	14 (7.1%)	58 (7.8%)	0.83
CDK12	14 (7.1%)	42 (5.7%)	0.57
BRAF	13 (6.6%)	32 (4.3%)	0.26
ATM	12 (6.1%)	48 (6.5%)	0.95
CTNNB1	11 (5.6%)	36 (4.9%)	0.84
FGFR1	11 (5.6%)	26 (3.5%)	0.27
EGFR	11 (5.6%)	24 (3.2%)	0.19
CCND1	11 (5.6%)	17 (2.3%)	0.03
PTEN/TP53/RB1 co-mutations			
Any co-mutation	26 (13.1%)	133 (18.0%)	0.13
TP53/PTEN	18 (9.1%)	106 (14.3%)	0.07
PTEN/RB1	7 (3.5%)	30 (4.1%)	0.9
TP53/RB1	9 (4.5%)	35 (4.7%)	1.0

Abbreviations: mCRPC, metastatic castration-resistant prostate cancer

© 2023 Hwang C et al. *JAMA Network Open*

eFigure 3. Oncoprint of Black and White Cohorts

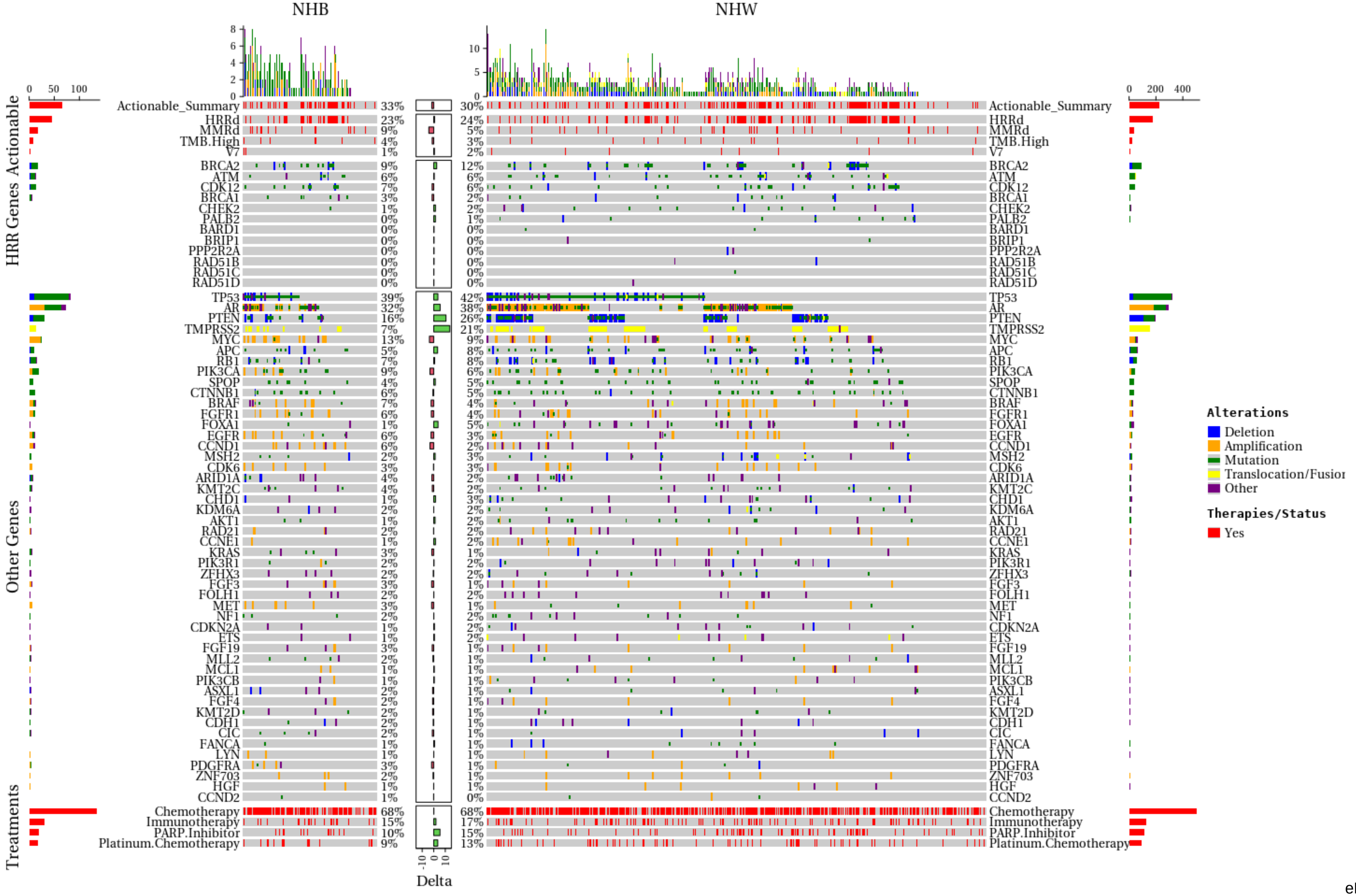

eFigure 3

Actionable molecular data, HRR gene alterations, other genetic alterations, and clinical treatment information are presented for each patient. Items within each category are ordered by % frequency across all patients. Patients are separated based on race (Black vs White) and % frequency is indicated in the middle column. The upper track indicates the number of gene alterations per patient and the side tracks indicate the number of alterations per gene, with colors indicating the type of alteration (blue for deletion, orange for amplification, green for mutation, yellow for translocation, and purple for others) or whether a status or treatment was present (red for present). Abbreviations: HRR, homologous recombination repair; MMRd, mismatch repair deficiency; TMB, tumor mutational burden; PARP, poly-ADP ribose polymerase.

© 2023 Hwang C et al. *JAMA Network Open*

eTable 2. Response Rates to Biomarker Directed Therapy

	Black	White	p-value
**Immunotherapy**			
PSA response rate	100.0% (n=3)	66.7% (n=18)	0.62
Radiographic response rate	50.0% (n=4)	61.5% (n=13)	1
**PARP inhibitor**			
PSA response rate	30.8% (n=13)	41.9% (n=62)	0.66
Radiographic response rate	22.2% (n=9)	14.8% (n=61)	0.93
**Platinum**			
PSA response rate	66.7% (n=6)	42.0% (n=50)	0.48
Radiographic response rate	25.0% (n=4)	31.6% (n=38)	1

Abbreviations: PSA, prostate-specific antigen; PARP, poly-ADP ribose polymerase

© 2023 Hwang C et al. *JAMA Network Open*
