# Supplementary material for: Biomarker-Directed Therapy in Black and White Men With Metastatic Castration-Resistant Prostate Cancer
Source: JAMA Netw Open. 2023 Sep 18;6(9):e2334208. doi: 10.1001/jamanetworkopen.2023.34208 (PMC10507489; doi:10.1001/jamanetworkopen.2023.34208)
Supplement: Supplement 2. — Data Sharing Statement [file jamanetwopen-e2334208-s002.pdf]

Data Sharing Statement

Hwang. Biomarker-Directed Therapy in Black and White Men With Metastatic Castration-Resistant Prostate Cancer. *JAMA Netw Open*. Published September 18, 2023. doi:10.1001/jamanetworkopen.2023.34208

Data
Data available: Yes
Data types: Deidentified participant data, Data dictionary
How to access data: contact Ajjai Alva, MD Clinical Professor Division of Hematology and Oncology Department of Internal Medicine Rogel Cancer Center 1500 E. Medical Center Drive Ann Arbor, Michigan 48109-5912 ajjai@med.umich.edu
When available: With publication

Supporting Documents
Document types: None

Additional Information
Who can access the data: Upon request and per consortium guidelines
Types of analyses: Upon request and per consortium guidelines
Mechanisms of data availability: per consortium guidelines with data access agreement
